# Effects of Saccharin Consumption on Operant Responding for Sugar Reward and Incubation of Sugar Craving in Rats

**DOI:** 10.3390/foods9121823

**Published:** 2020-12-08

**Authors:** Kenjiro Aoyama, Akane Nagano

**Affiliations:** Department of Psychology, Doshisha University, Kyotanabe-shi, Kyoto 610-0394, Japan; anagano@mail.doshisha.ac.jp

**Keywords:** artificial sweetener, saccharin, sucrose, body weight gain, operant conditioning, incubation of craving, conditioned satiety response, rats

## Abstract

Repeated experience with artificial sweeteners increases food consumption and body weight gain in rats. Saccharin consumption may reduce the conditioned satiety response to sweet-tasting food. Rats were trained to press a lever to obtain sucrose for five days. A compound cue (tone + light) was presented with every sucrose delivery. On the following day, each lever press produced only the compound cue (cue-reactivity test). Subjects were then provided with yogurt for three weeks in their home cages. The rats were divided into two groups. Rats in the saccharin group received yogurt sweetened with saccharin on some days and unsweetened yogurt on others. For the plain group, only unsweetened plain yogurt was provided. Subsequently, the cue-reactivity test was conducted again. On the following day, the rats underwent a consumption test in which each lever press was reinforced with sucrose. Chow consumption and body weight gain were larger in the saccharin group than in the plain group. Lever responses increased from the first to the second cue-reactivity tests (incubation of craving) in both groups. During the consumption test, lever responses were higher in the saccharin group than in the plain group, suggesting that the conditioned satiety response was impaired in the saccharin group.

## 1. Introduction

### 1.1. Paradoxical Effects of Artificial Sweeteners on Body Weight Gain in Rodents

Non-caloric, artificial sweeteners such as saccharin, aspartame, sucralose, or acesulfame potassium are frequently used in a wide variety of foods and drinks. People choose these artificial sweeteners to reduce their body weight. However, a paradoxical relationship between the consumption of artificial sweeteners and body weight has been shown in pioneering experimental studies conducted by investigators at Purdue University. They demonstrated that these sweeteners could cause various health problems, such as overeating and excessive weight gain, in rats [1,2,3]. Other research groups have replicated and extended these results [4,5,6,7,8].

One plausible mechanism to explain such paradoxical effects of artificial sweeteners focuses on the role of Pavlovian conditioning. Studies have shown that Pavlovian conditioning contributes to the regulation of energy control [9,10]. Investigators at Purdue University extended these ideas to the effects of artificial sweetener on the regulation of energy intake and body weight [11,12,13]. According to their hypothesis, when animals consume foods containing sugar, they experience a predictive relationship between a sweet taste and its postingestive consequences (e.g., the arrival of energy in the gut). With the repeated experience of this predictive relationship, the sweet taste becomes a conditioned stimulus (CS) for postingestive unconditioned stimulus (US). Consequently, the taste CS begins to elicit conditioned responses (CRs), which often contributes to the termination of feeding behavior. The termination of feeding behavior arising from these CRs is termed the "conditioned satiety response", and the importance of conditioned satiety in the regulation of feeding behavior is well documented [14,15,16,17]. However, the ingestion of foods containing artificial sweeteners can impair the predictive nature of the relationship, because the anticipated consequences no longer accompanied the sweet taste. Therefore, repeated experience with artificial sweeteners may impair an animal’s ability to respond appropriately to sweet taste. Consequently, when food containing real sugar is actually consumed, the conditioned satiety response to the sweet taste may be slower and/or smaller.

A few studies [1,12] have tested whether artificial sweeteners experience impaired conditioned satiety response to sweet-tasting food. In these studies, rats in one group repeatedly experienced artificial sweeter (saccharin), but rats in the other group did not experience artificial sweeter. In the following test, rats were fed a novel calorically sweetened premeal immediately before the measurement of chow consumption. If the artificial sweeteners experience impairs the conditioned satiety response to sweet-tasting premeal, rats without the artificial sweetener experience should demonstrate caloric compensation for the sweet-tasting premeal by reducing subsequent chow consumption. In contrast, rats with artificial sweeteners experience should not. The results confirmed this prediction. Thus, the studies that examined the satiating effect of sweet-tasting food on subsequent chow consumption provided evidence to support the Pavlovian condition hypothesis.

### 1.2. Effects of Repeated Consumption of Artificial Sweetener on Sucrose Consumption

The Pavlovian conditioning hypothesis also predicts that repeated consumption of artificial sweeter should impair the conditioned satiety response to sweet-tasting food, and thus increase consumption of the sweet-tasting food "itself". However, this prediction has not yet been empirically examined. The first purpose of this study was to therefore test this prediction. In the present study, subjects were first trained to press a lever in an operant conditioning chamber to obtain sugar pellets. Half of the subjects (plain group; PLA group) were then fed plain, unsweetened yogurt (~0.6 kcal/g) in home cages for six days per week, during a three-week yogurt period. The other subjects (saccharin group; SAC group) were fed plain yogurt for three days per week and yogurt sweetened with saccharin (~0.6 kcal/g) in home cages for three days per week during the three weeks. After the three-week yogurt period, all subjects were tested for 30 min in the operant chamber. If the consumption of artificial sweetener impairs the conditioned satiety response to the sweet-tasting food, the number of lever responses for sugar pellets should be larger in the SAC than in the PLA group. This is the first research hypothesis in this study. Additionally, in order to examine the effects of the impairment of the conditioned satiety response to sweet-tasting food closely, the within-session decreases (time-course) of operant responses for sugar pellets were investigated. Analysis of within-session changes in operant response helps to explore factors in controlling operant behavior [18]. According to the Pavlovian conditioning hypothesis, consumption of each sugar pellet should produce less conditioned satiety response in the SAC group than in the PLA group. Thus, within-session decreases of operant responses should be shallower in the SAC group than in the PLA group.

### 1.3. Effects of Repeated Consumption of Artificial Sweetener on Incubation of Sugar Craving

The second purpose of the present study was to investigate the effect of repeated consumption of artificial sweeteners during a forced-abstinence period of sugar on the incubation of sugar craving. Cue-induced seeking behavior (cue-reactivity) is often used as a rodent model of craving [19,20]. Incubation of craving has been observed in rats with repeated experiences of sucrose self-administration [21,22,23,24]. In these studies, subjects were first trained to press a lever to obtain sucrose solution for successive daily sessions, in which each sucrose presentation was paired with a discrete compound cue (tone + light) and then tested after forced-abstinence of sucrose. During the test, each lever response was accompanied only by the compound cue. Responses to the cue were enhanced after prolonged abstinence. Examining the abstinence-dependent growth in cue-reactivity could unveil treatment strategies for chronic relapsing disorders such as obesity and eating disorders.

Recently, it has been demonstrated that responses to a cue associated with saccharin were also enhanced after forced-abstinence from saccharin [25]. This result suggests that a postingestive caloric consequence is not a necessary condition, and sweet taste itself is an important factor for the growth of incubation of sugar craving. Accordingly, when non-caloric artificial sweeteners were provided repeatedly during the forced-abstinence period of sugar, incubation of sugar craving could be weakened. This prediction has not yet been examined. Cue-reactivity tests were performed before and after the three-week yogurt period to test this prediction. In the PLA group, all subjects were deprived of sweet-tasting foods for the entire yogurt period. In the SAC group, subjects were deprived of sugar, but sweet-tasting food (i.e., yogurt sweetened with saccharin) was provided during this period. Therefore, the incubation of sugar craving may be smaller in the SAC group than in the PLA group. This is the second research hypothesis in this study.

## 2. Materials and Methods

### 2.1. Subjects

The subjects were 14 naive male albino rats bred from Wistar stock. The rats were approximately 80 days old, weighing 282–313 g at the start of the experiment. They were housed individually in wire-mesh home cages (24.0 × 20.0 × 20.0 cm high). Nutritionally balanced rodent chow (LabDiet 5001, 3.02 kcal/g) was available ad libitum except as noted below. Water was always available in the home cage. The animal room was maintained on a 12-h/12-h light/dark cycle (lights on at 8 a.m.). Before the first day of the self-administration (SA) training, rodent chow was removed for 17 h to promote the acquisition of lever-press responses. Rodent chow was then returned to their home cages. All procedures were approved and performed in accordance with the guidelines of the Animal Research Committee of Doshisha University (authorization number A16006, Kyotanabe, Kyoto, Japan).

### 2.2. Apparatus

Operant training and testing took place in conditioning chambers (24.0 × 31.0 × 21.0 cm high; Med Associates). The front wall of the chamber contained the food opening and two retractable levers (4.5 × 0.1 cm) that extended 1.8 cm into the chamber. Each lever was located 1.0 cm from the side wall and 2.0 cm from the floor. Only the left lever was used in this experiment. A rectangular food opening (5.0 × 4.5 cm) was located 9.5 cm from the side wall and 0.5 cm from the floor. The front wall also contained two white cue lights above the levers and a tone generator (2 kHz, 15 dB over ambient noise) above the left lever. Only the white cue light above the left lever was used in this experiment. A red house light (24-V) was located in the back wall and 2.0 cm below the ceiling. Operant chambers were contained in sound-attenuating enclosures. A ventilating fan masked noises from outside the enclosures.

### 2.3. Procedure

**Figure 1:**
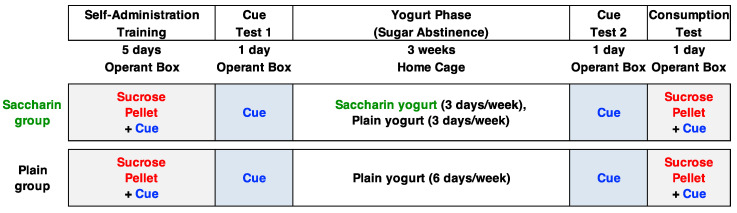
General experimental structure. All subjects received the same training and tests. During the yogurt phase, subjects in the saccharin (SAC) group received plain, unsweetened yogurt for three of the six days each week, and received yogurt sweetened with saccharin for the other three days. Subjects in the plain (PLA) group received plain, unsweetened yogurt for six of the six days each week.

The general experimental structure is shown in Figure 1. Procedures for self-administration training, cue-tests, and consumption test were generally based on previous studies about the incubation of sugar craving [22,23,24,25], and the procedures for the yogurt phase were generally based on previous studies from the Purdue group [1,2,3]; however, details were adjusted for this study.

#### 2.3.1. Self-Administration Training Phase

Rats were trained to press a lever in the operant chamber for five consecutive days. They spent 1 h per day for the first two days and 30 min per day for the remaining three days in the chambers. Each lever-press response was reinforced by a 45 mg sucrose pellet (Bio Serv^®^ Dustless Precision Pellets^®^, Sugar, Product #F0042, Bio Serv, Flemington, New Jersey, USA). A lever response was also accompanied by a compound cue consisting of the white cue light and tone. The duration of the compound cue was three seconds. The red house light was lit throughout the self-administration (SA) training sessions. The subjects were returned to their home cages at the end of each session.

#### 2.3.2. Cue-Reactivity Test 1

On the day following the last day of SA training, rats were tested in the operant chambers for sugar cue-reactivity (sugar seeking). This session was identical to the 30-min SA training procedure, but the sucrose pellet did not accompany the lever response.

#### 2.3.3. Yogurt Phase

At the end of cue-reactivity test 1, rats (*n* = 7 rats/group) were divided into two groups (saccharin [SAC] and plain [PLA] groups). Lever-press responses in the SA training phase and cue-reactivity test 1 were matched between groups. During the 3-week yogurt phase, rats were housed in home cages and received 20 g of yogurt (Meiji, Tokyo, Japan) daily for six days per week in addition to ad libitum rodent chow and water. Yogurt diets were provided in aluminum cups attached to the inside of the home cage. The cups were left in the cage for approximately 23 h per day. On the 7th day of each week, only rodent chow and water were provided and the aluminum cups were not attached to the cage. No sucrose was provided during the yogurt period. Yogurt and chow intake were recorded daily. Rats in the SAC group received yogurt sweetened with 0.3% saccharin (*wt*/*wt*; ~0.6 kcal/g) for three of the six days each week and received plain, unsweetened yogurt (~0.6 kcal/g) for the other three days that week. The presentation order of the yogurt was randomized each week. Conversely, rats in the PLA group were provided with plain, unsweetened yogurt for six days each week.

#### 2.3.4. Cue-Reactivity Test 2

On the day following the last day of the yogurt phase, a second cue-reactivity test was conducted. The procedure was identical to the cue-reactivity test performed before the yogurt period.

#### 2.3.5. Consumption Test

The next day, a consumption test was conducted. The procedure of the consumption test was identical to the 30-min training; each lever-press was accompanied by a sucrose pellet and the compound cue.

### 2.4. Statistical Analyses

#### 2.4.1. Self-Administration Training Phase

The average number of lever-press responses for the last three days of the SA training phase was compared between the SAC and PLA groups using a *t*-test.

#### 2.4.2. Yogurt Phase

The weight of consumed yogurt was averaged over the entire yogurt phase and compared between the SAC and PLA groups using a *t*-test. Body weight on the first day of the yogurt phase was also compared between groups using a *t*-test. Body-weight gain and chow intake were analyzed separately by a two-way analysis of variance (ANOVA) using WEEKS as the within-group factor (weeks 1 to 3) and GROUP (SAC or PLA) as the between-groups factor.

#### 2.4.3. Cue-Reactivity Tests

The number of lever-press responses during cue reactivity tests 1 and 2 were analyzed by a two-way ANOVA with CUE-TESTS as the within-group factor (Test 1 or Test 2) and GROUP (SAC or PLA) as the between-groups factor.

#### 2.4.4. Consumption Test

The number of lever-press responses during the consumption test was compared between the SAC and PLA groups using a *t*-test. One rat in the SAC group was excluded from the analysis due to a problem with the apparatus.

#### 2.4.5. Within-Session Changes in Lever-Press Responses during the Consumption Test

The 30-min consumption test session was divided into six 5-min blocks. First, group response rates (number of lever-press responses per 5 min) were described as a function of time blocks. Within-session changes in lever-press responses were analyzed using a two-way ANOVA with TIME-BLOCKS as the within-group factor (blocks 1 to 6) and GROUP (SAC or PLA) as the between-groups factor.

Second, response rates were described as a function of the cumulative number of sucrose pellet deliveries. Previous studies have shown that rates of operant responses decrease proportionally with the increase in the cumulative number of reinforcer deliveries [25,26,27,28]. These studies [25,26,27,28] have demonstrated that the relationship can be described by Equation (1).

(1) Rr=a−b Ic

As mentioned in previous studies [25,26,27,28], *Ic* is the cumulative number of reinforcer deliveries, *a* and *b* are free parameters, and *Rr* is the response rate. The response rate represents the number of responses per unit time. Parameter *a* indicates the *y*-axis intercept of the regression line, representing the response rate at the start of the session. Parameter *b* indicates the slope of the regression line, representing the rate of response decrease produced by consumption of each reinforcer. Another parameter can be derived from Equation (1): the *x*-axis intercept of the regression line, predicting the cumulative number of reinforcer deliveries that will reduce the response rate to zero. Equation (1) was applied separately for the SAC and PLA groups. Equation (1) was also applied to each rat. A *t*-test was used to compare the parameters (i.e., *y*-axis intercept, *x*-axis intercept, slope, and *r*^2^) between the SAC and PLA groups.

ANOVAs and *t*-tests were performed using SPSS Statistics version 25.0. Group data are presented as mean ± standard error of the mean (*SEM*). Significance levels were set at *p* < 0.05. Data of this study are available in the Supplementary Materials (Table S1: Data).

## 3. Results

### 3.1. Self-Administration Training Phase

The number of lever-press responses averaged for the last three days of the training phase was 42.2 ± 6.7 for the SAC group and 43.8 ± 6.9 for the PLA group. There was no significant difference between the groups, *t* (12) = 0.16, *p* = 0.87, *d* = 0.09.

### 3.2. Yogurt Phase

During the yogurt phase, almost all yogurt was consumed in the home cages. The average yogurt consumption per day was 19.9 ± 0.01 g for the SAC group and 19.9 ± 0.01 g for the PLA group. Yogurt consumption did not differ between groups, *t* (12) = 0.85, *p* = 0.41, *d* = 0.38. The average body weight on the first day of the yogurt phase did not differ between the SAC (326.6 ± 2.9 g) and PLA (328.1 ± 5.5 g) groups, *t* (12) = 0.25, *p* = 0.80, *d* = 0.13. Chow intake during the two days before yogurt phase (i.e., from just after the last session of the self-administration training to just before the first presentation of yogurt) did not differ between the SAC (54.7 ± 2.8 g) and PLA (58.1 ± 2.6 g) groups, *t* (12) = 0.90, *p* = 0.39, *d* = 0.48. Thus, body weight and chow consumption were comparable at the beginning of the yogurt phase.

**Figure 2:**
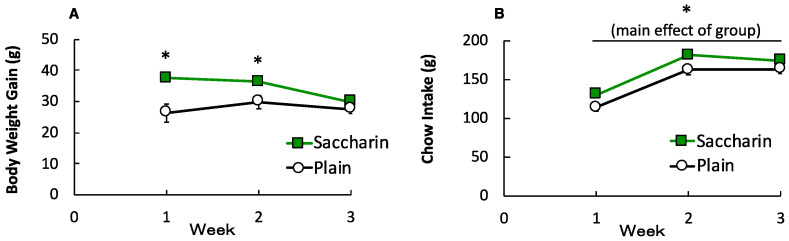
Mean body weight gain (**A**) and mean chow consumption (**B**) during the yogurt phase. Data points indicate means ± SEMs (*n* = 7 for each group). The error bars were sometimes smaller than the height of the symbols. Asterisk indicates a significant difference between the groups.

Figure 2A depicts the body weight gain across three weeks. A two-way ANOVA revealed a significant main effect of GROUP, *F* (1, 12) = 9.46, *p* = 0.01, *η_p_*^2^ = 0.44, indicating that consumption of saccharin-sweetened yogurt facilitated body-weight gain. A significant main effect of WEEKS, *F* (2, 24) = 5.44, *p* = 0.01, *η_p_*^2^ = 0.31, and a significant interaction of GROUP and WEEKS was also found, *F* (2, 24) = 5.06, *p* = 0.02, *η_p_*^2^ = 0.30. Analysis of simple main effects revealed significant differences between the SAC and PLA groups in weeks 1 and 2 but not in week 3. Figure 2B depicts the chow consumption across 3 weeks. A two-way ANOVA showed a significant main effect of GROUP, *F* (1, 12) = 6.18, *p* = 0.03, *η_p_*^2^ = 0.34, indicating that the presentation of saccharin-sweetened yogurt facilitated chow consumption. A main effect of WEEKS was also statistically significant, *F* (2, 24) = 395.34, *p* = 0.001, *η_p_*^2^ = 0.97. An interaction of GROUP and WEEKS was not statistically significant, *F* (2, 24) = 1.88, *p* = 0.18, *η_p_*^2^ = 0.14.

### 3.3. Cue-Reactivity Tests

**Figure 3:**
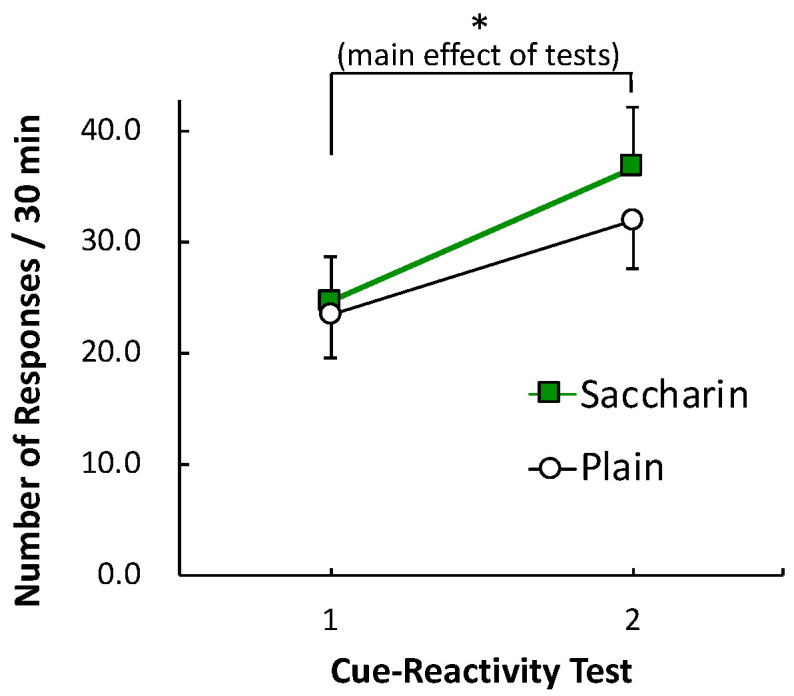
Mean number of lever-press responses in cue-reactivity tests 1 and 2. Data points indicate means ± SEMs (*n* = 7 for each group). Asterisk indicates a significant difference between the tests.

Figure 3 represents the average number of lever-press responses in the cue-reactivity tests 1 and 2. A two-way ANOVA revealed a significant effect of CUE-TESTS (Test 1 or Test 2), *F* (1, 12) = 18.03, *p* = 0.001, *η_p_*^2^ = 0.60, indicating an “incubation of craving.” However, a main effect of GROUP, *F* (1, 12) = 0.27, *p* = 0.61, *η_p_*^2^ = 0.02, and an interaction of GROUP and CUE-TESTS, *F* (1, 12) = 0.50, *p* = 0.49, *η_p_*^2^ = 0.04, were not significant. Thus, consumption of saccharin-sweetened yogurt intermittently during the 3-week yogurt period did not attenuate the incubation of sucrose craving.

### 3.4. Consumption Test

**Figure 4:**
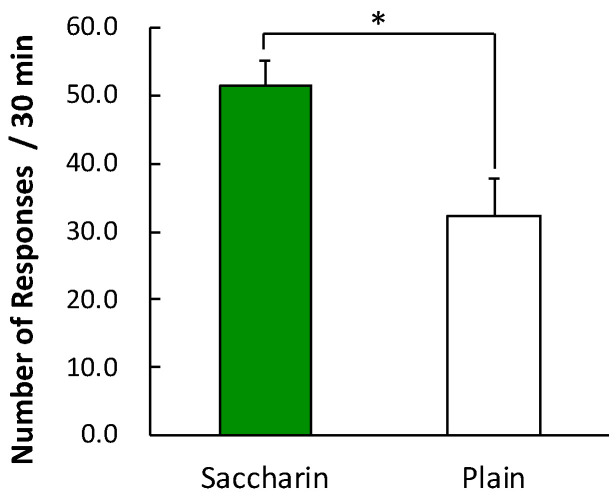
Mean number of lever-press responses in the consumption test. Means ± SEMs are indicated in the figure (*n* = 6 for the SAC group and *n* = 7 for the PLA group). Asterisk indicates a significant difference between the groups.

Figure 4 presents the average number of lever-press responses in the consumption test. The SAC group pressed the lever more than the PLA group, *t* (11) = 2.69, *p* = 0.02, *d* = 1.50. Thus, the repeated consumption of saccharin facilitated the subsequent consumption of sucrose pellets.

### 3.5. Within-Session Changes in Lever-Press Responses during the Consumption Test

**Figure 5:**
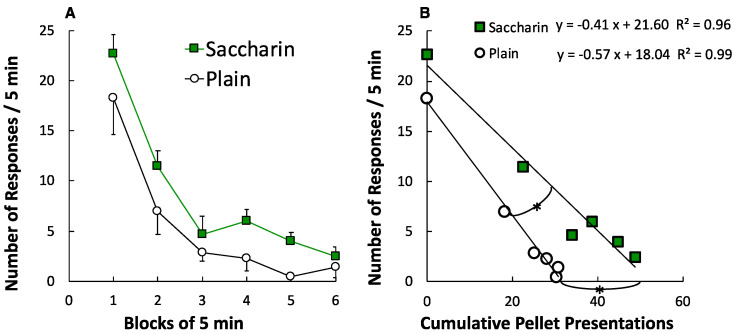
Time courses of lever-press responses in the consumption test. (**A**) Mean response rates (number of lever-press responses per 5 min) as a function of successive 5-min blocks. Data points indicate means ± SEMs (*n* = 6 for SAC group and *n* = 7 for PLA group). (**B**) Mean response rates as functions of cumulative number of sucrose pellet deliveries. Each data point represents the average of the cumulative number of pellet deliveries at the beginning of a 5-min block, and the average of the response rate of that 5-min block. Asterisk indicates a significant difference between the groups in the parameters of Equation (1).

Figure 5A shows the mean response rate (number of lever-press responses per 5 min) as a function of successive 5-min blocks. Lever-press responses decreased during the 30-min consumption test with TIME-BLOCK, *F* (5, 55) = 42.34, *p <* 0.001, *η_p_*^2^ = 0.79. In addition, the SAC group pressed the lever significantly more than the PLA group with GROUP, *F* (1, 11) = 7.22, *p* = 0.02, *η_p_*^2^ = 0.40. The interaction between TIME-BLOCK and GROUP was not significant, *F* (5, 55) = 0.42, *p* = 0.84, *η_p_*^2^ = 0.04.

Figure 5B presents the mean response rates as functions of the cumulative number of sucrose pellet deliveries. The linear relationship between the response rate and the cumulative number of pellet deliveries was evident in both groups, *r*^2^s > 0.96. Table 1 indicates the parameters of Equation (1) when it was applied to individual rats. The *x*-intercept of the regression line for the SAC group was larger than that for the PLA group, *t* (11) = 2.76, *p* = 0.02, *d* = 1.53. Moreover, the slope of the regression line for the SAC group was shallower than that for the PLA group, *t* (11) = 2.55, *p* = 0.03, *d* = 1.42. For *y*-axis intercepts and *r*^2^, the differences were not significant, *t* (11) = 0.93, *p* = 0.37, *d* = 0.53, and *t* (11) = 0.38, *p* = 0.71, *d* = 0.21, respectively.

## 4. Discussion

### 4.1. Effects of Saccharin Experience on the Consumption of Sweet-Tasting Food

The first purpose of the present study was to investigate the effects of artificial sweetener consumption on the subsequent intake of sweet-tasting food. Rats in the SAC group showed more operant responses in obtaining sugar pellets than those in the PLA group. This result is consistent with the first research hypothesis. According to the Pavlovian conditioning hypothesis, repeated consumption of artificial sweeteners should impair the conditioned satiating effect of sweet-tasting food, and thus increase consumption of the sweet-tasting food itself. Additionally, within-session changes of operant responding were analyzed. Equation (1) adequately described within-session decreases in responding, as shown in several previous studies [25,26,27,28]. More importantly, the analysis revealed that the slope of the regression line was less steep in the SAC group than in the PLA group. The slope indicates the rate of response decrease produced by the consumption of each sugar pellet. If repeated consumption of artificial sweetener impairs the conditioned satiety response to the sugar pellet, the slope for the SAC group should be less steep than that for the PLA group. Therefore, the analysis of within-session patterns also provided evidence to support the Pavlovian conditioning hypothesis.

As mentioned in the introduction, a few studies [1,12] have shown the effects of artificial sweeter experience on the conditioned satiety response from a caloric compensation perspective. However, their results cannot rule out another interpretation. In one study [1], rats in one group were provided with unsweetened yogurt for seven days and saccharin-sweetened yogurt for the remaining seven days. In the other group, rats received unsweetened yogurt for seven days and yogurt sweetened with caloric sweetener (glucose) for the remaining seven days. Thus, only the latter group had experienced caloric sweeteners. The experience of caloric sweeter could facilitate the conditioned satiety response to sweet-tasting food. Therefore, the observed difference in response to sweet-tasting premeal could have resulted from the facilitation of conditioned satiety response in the latter group rather than the impairment of conditioned satiety response in the former group. In another study [12], rats in one group received the saccharin-sweetened solution for five days and the glucose sweetened solution for the remaining five days. In the other group, rats were provided with the glucose-sweetened solution for five days and the sucrose-sweetened solution for the remaining five days. Thus, the latter group had more experience of caloric sweeter than the former group. Therefore, the facilitated conditioned satiety response in the latter group could explain the results in the same way. In the present study, caloric sweeteners were not provided during the yogurt phase in either group. Thus, the results of the consumption test cannot be explained by the facilitated conditioned satiety response induced by caloric sweetener experience. Consequently, the results of the present study strengthened the hypothesis that artificial sweetener experience impaired conditioned satiety response to sweet-tasting food.

### 4.2. The Relathionship betweeen the Impairment of the Conditioned Satiety Response and the Body Weight Gain

Although the consumption test results indicated that consumption of artificial sweeteners impaired the conditioned satiety response, it does not mean that the reduction in conditioned satiety response is a critical factor facilitating body weight gain. As seen in Figure 2A, the difference in body weight gain between the SAC and PLA groups disappeared in the last week of the yogurt phase. The Pavlovian conditioning hypothesis cannot fully explain the results of the body weight gain, because the attenuation of the taste CS-postingestive US association is supposed to be the largest in the last week. Therefore, factors other than impairment in conditioned satiety may contribute to the facilitation of body weight gain. Some studies have shown that consumption of artificial sweeteners increases body weight gain without affecting total caloric intake [5,6,7,8]. These results cannot be explained by the reduction in the conditioned satiety response. The researchers in those studies claimed that artificial sweeteners promote dysregulation of body weight by reducing energy expenditure [7]. Additionally, the consumption of artificial sweeteners may also impair body weight regulation via changes in the intestinal microbiota. Studies in rodents have shown increases in body weight associated with alterations in the balance of gut bacteria in response to artificial sweeteners [29,30,31]. Multiple factors appear to be involved in body weight gain induced by artificial sweetener consumption.

As stated above, several studies have reported the effects of artificial sweeter consumption on body weight gain [1,2,3,4,5,6,7,8,29,30,31]. However, the consumption of artificial sweeteners does not always facilitate body weight gain. One recent study failed to find any accelerating effects on chow consumption and body weight gain by artificial sweeteners [32]. According to the Pavlovian conditioning hypothesis, repeated consumption of artificial sweeteners should degrade the animal’s capability to respond appropriately to sweet-tasting food. Therefore, the sweetness of the chow may be a critical factor in determining the effects of artificial sweeteners. The article that failed to find the effect of artificial sweetener [32] reported that the free sugar content of the chow (Rat and Mouse Cubes, Specialty Feeds) used in their study was approximately 1.3%. In comparison, the chow (LabDiet 5001) used in the study by the Purdue group [1] contains approximately 4% sugars. The present study used the latter chow. Therefore, the manifestation of the artificial sweeteners’ undesirable effect on body weight may require a level of sweetness in the maintenance diet (i.e., chow). However, another recent study [33] reported that mice offered water sweetened with artificial sweetener did not significantly increase body weight than the group consuming water without sweetener when fed high-fat high-sucrose maintenance diets. Thus, the sweetness of the maintenance diet could not be the single factor that determines the manifestation of the effects of artificial sweeteners on body weight.

### 4.3. Effects of Saccharin Consumption on Incubation of Sugar Craving

The second purpose of the present study was to investigate the effect of repeated consumption of artificial sweeteners during a forced-abstinence period of sugar on the incubation of sugar craving. In both groups, the number of lever-press responses in the second cue-reactivity test was larger than that in the first cue-reactivity test. Thus, incubation of sugar craving was observed, similar to the results of previous studies [21,22,23,24]. However, the increase in lever responses from cue-reactivity test 1 to cue-reactivity test 2 was similar in the SAC and PLA groups. Thus, repeated consumption of artificial sweeter during the three-week forced sugar abstinence period did not attenuate the incubation of sugar craving. The results did not support the second research hypothesis. Previous studies showed that overnight access to sucrose on the previous day of the cue-reactivity test did not attenuate the development of incubation of craving [23,34]. Results from these studies and the present study suggest that neither ad libitum access to sweeteners at the end of the abstinent period, nor intermittent access to sweeteners during the abstinent period were sufficient to inhibit the development of incubation of craving. Further studies are needed to explore the factors that could attenuate the incubation of sugar craving.

### 4.4. Conclusions

As shown in the present study, chow intake and body weight gain were higher in the SAC group than in the PLA group. Additionally, the number of lever responses for sugar pellets was larger in the SAC group than in the PLA group. Moreover, within-session decreases in operant responding were slower in the SAC group than in the PLA group. These results support the hypothesis that consumption of artificial sweeteners reduces the conditioned satiety response to sweet-tasting food. The generality of findings, from rats in their highly controlled settings to humans in their complicated environments, should be considered carefully. However, results obtained from animal experiments designed to reveal the causal relationship are consistent with human epidemiological studies that showed a correlation between heightened consumption of artificial sweeteners and heightened prevalence of overweight and obesity [35,36,37,38]. Conversely, recent reviews suggest that randomized controlled trials in humans generally demonstrate the benefits of artificial sweetener use on body weight, particularly when used alongside behavioral weight loss support [39,40,41]. Identifying a plausible mechanism by which an artificial sweeter may affect body weight gain is important for providing efficient behavioral weight loss support.

## Figures and Tables

**Table 1:** Individual subject parameters derived from Equation (1).

Saccharin Group					Plain Group				
Subjects *	*y*-axis	*x*-axis	Slope	*r* ^2^	Subjects *	*y*-axis	*x*-axis	Slope	*r* ^2^
A18	27.21	47.46	0.573	0.98	A17	22.18	47.64	0.466	0.87
A23	18.74	63.22	0.296	0.87	A20	21.95	23.84	0.921	0.99
A24	25.34	70.99	0.357	0.91	A21	23.08	53.82	0.429	0.90
A25	24.90	50.36	0.494	0.91	A22	15.76	27.46	0.574	0.89
A27	22.17	48.24	0.460	0.91	A29	13.37	24.32	0.550	0.74
A28	12.89	39.94	0.323	0.74	A30	7.01	8.03	0.874	0.99
					A32	27.16	38.53	0.705	0.96

* The columns show ID number for each subject.

## Supplementary Materials

The following is available online at https://www.mdpi.com/2304-8158/9/12/1823/s1, Table S1: Data.
